# Alcohol Consumption and Beverage Preferences in a Predominantly Female, Highly Educated Spanish Population: A Sociodemographic and Network Analysis

**DOI:** 10.3390/foods14111930

**Published:** 2025-05-29

**Authors:** Elena Sandri, Michela Capoferri, Gaia Luciani, Michela Piredda

**Affiliations:** 1 Faculty of Medicine and Health Sciences, Catholic University of Valencia San Vicente Mártir, c/Quevedo 2, 46001 Valencia, Spain; 2 Doctoral School, Catholic University of Valencia San Vicente Mártir, c/Quevedo 2, 46001 Valencia, Spain; 3 Department of Animal Production and Health, Veterinary Public Health, and Food Science and Technology, Faculty of Veterinary Medicine, Institute of Biomedical Sciences, Cardenal Herrera-CEU University, CEU Universities, Calle Santiago Ramón y Cajal 20, 46115 Alfara del Patriarca, Spain; michelacapoferri77@gmail.com; 4 Department of Biomedicine and Prevention, Tor Vergata University, Via Montpellier 1, 00133 Rome, Italy; gaia.luciani.gl@gmail.com; 5 Research Unit Nursing Science, Department of Medicine and Surgery, Campus Bio-Medico di Roma University, Via Alvaro del Portillo 21, 00128 Rome, Italy

**Keywords:** alcoholic beverages, fermented beverages, sociodemographic factors, survey, Spain

## Abstract

Understanding alcohol consumption patterns is critical for developing effective public health strategies, particularly in countries like Spain where cultural and regional drinking norms vary widely. This study examined sociodemographic factors affecting alcohol consumption patterns across Spain, employing a cross-sectional design. A total of 22,181 Spanish adults over 18 years of age were recruited between August 2020 and November 2021, using non-probabilistic snowball sampling through social media networks. Data were gathered via a validated questionnaire (NutSo-HH Scale) encompassing sociodemographic details, health indicators, and lifestyle habits, with a focus on alcohol use. The sample included *n* = 22,181 participants, 80.8% women, with a mean age of 34.9 years. Most respondents (48.2%) reported no or very occasional alcohol consumption, 33% drank 2–4 times per month, 13.8% consumed alcohol 2–3 times weekly, and 5% drank daily or nearly daily. Alcohol consumption was significantly higher among men (72.1% consuming fermented beverages) and individuals with higher income and education (*p* < 0.001 for all variables). Regional differences were also notable, with the highest percentage of regular drinkers in Asturias (80.9%) and the Valencian Community (73.3%) as revealed by a Kruskal–Wallis test (*p* < 0.001). Fermented beverages were the most popular, with 68.4% of alcohol consumers preferring these, compared to distilled beverages (18.8%), fortified beverages (15.1%), and liqueurs (3.3%). A Gaussian graphical model was used to explore conditional relationships between alcohol consumption and other beverages in the Spanish population. Alcohol showed strong positive associations with fermented and distilled beverages, and with the habit of getting drunk. Weaker negative correlations were observed with water and soft drinks, suggesting contrasting consumption patterns. These findings underscore significant sociodemographic and regional variations in alcohol consumption patterns across Spain, suggesting the need for public health interventions tailored to different population segments.

## 1. Introduction

As a beverage with a rich historical background, alcohol has fluctuated between being celebrated and condemned through the ages. In contemporary society, it represents a widely debated topic in public health and social discourse, subjected to rigorous monitoring with guidelines and limits established by organizations such as the World Health Organization (WHO) [1]. Patterns of consumption are shaped by cultural norms, social contexts, and individual identities and vary significantly across sociodemographic and regional groups in Spain [2]. Often associated with leisure, escapism, and enjoyment, its use nonetheless involves engagement with a substance that, although socially accepted, can be addictive and harmful [3]. Despite this rich cultural and scientific background, alcohol consumption remains a significant public health concern in Spain [4,5]. High rates of regular intake and changing social norms underscore the need to understand the factors influencing drinking behavior. Its consumption is pivotal in Spanish culture, intricately linked to social interactions, traditions, and culinary practices that form an integral part of daily life [6,7].

Spain’s diverse geography and climate have fostered the production of a broad range of alcoholic beverages with regionally distinct characteristics, particularly in viticulture, as exemplified by La Rioja, Ribera del Duero renowned for their red wines, and Rías Baixas celebrated for its Albariño white wine [8,9,10,11]. Sparkling wines like Catalonia’s Cava highlight Spain’s enological expertise, combining tradition with cultural heritage and playing a key role in celebrations and social gatherings [12,13]. A broad spectrum of alcoholic beverages is consumed in Spain, which can be categorized into four main types based on their production methods and characteristics [3].

Beer, wine, and cider are fermented beverages produced through the fermentation of carbohydrates found in grains, fruits, or other plant-based materials [14]. These drinks form an essential part of Spain’s cultural and gastronomic traditions, with wine and beer being particularly prominent. They account for a substantial share of alcohol consumption in the country, reflecting their central role in both traditional and modern social practices [15].

The category of distilled beverages includes, for instance brandy, gin, and rum, which are crafted by distilling fermented liquids to concentrate their alcohol content. A notable example is Brandy de Jerez, a product that highlights Spain’s enological expertise and heritage. Its production employs the traditional criaderas and soleras aging system, which imparts unique complexity and depth of flavor, underlining the historical importance of distilled beverages in Spain [16].

Fortified beverages, like sherry and port, are wines enhanced with distilled alcohol to increase their potency and shelf stability. These beverages hold a special place in Spain’s cultural and economic landscape, with Andalusia, particularly Jerez, being renowned for producing sherry. The unique climatic conditions and meticulous craftsmanship of this region contribute to the exceptional quality and global reputation of Spanish fortified wines [17].

Finally, liqueurs and creams like Licor de Hierbas and Crema de Orujo are distinct Spanish beverages, combining local botanicals with tradition and innovation and reflecting the country’s regional diversity and culinary ties [18].

Advances in microbiology and enology have significantly improved fermentation and aging processes in Spain, optimizing traditional practices such as yeast strain selection, temperature control, and barrel aging, thereby enhancing the complexity and consistency of beverages like Cava and Rioja wines [19]. Nevertheless, alcohol consumption continues to pose significant public health challenges in Spain. Understanding the drivers of consumption is critical, especially given the shifting cultural norms and persistent high intake levels. Moreover, gaps persist in the literature regarding how these patterns vary across different segments of the population. The sociodemographic factors influencing alcohol consumption in Spain are multifaceted. Elements such as age, gender, socioeconomic status, and regional identities play central roles in determining consumption patterns. For example, younger populations tend to opt for lighter beverages such as beers and cocktails, while older people may prefer traditional spirits or wines [20,21]. Socioeconomic determinants also impact consumption; economically disadvantaged groups may choose lower-cost alcoholic options, whereas those with higher incomes are more likely to favor premium wines and gourmet experiences [22].

Moreover, cultural attitudes toward alcohol consumption reflect broader social norms. In Spain, alcohol is often viewed as a social lubricant, integral to both interpersonal relationships and larger social frameworks. Celebratory events, religious festivals, and family gatherings frequently showcase a variety of beverages, reinforcing the role of alcohol in cultural cohesion [23]. This cultural acceptance can have both positive and negative implications for public health, highlighting the need for targeted interventions that respect cultural practices while encouraging responsible drinking. Prior studies on alcohol consumption in Spain have primarily emphasized its cultural, historical, and social significance, particularly focusing on traditional practices, production methods, and symbolic meanings of alcoholic beverages. However, less attention has been given to how sociodemographic and regional differences shape consumption patterns. There is a notable gap in research regarding alcohol consumption in Spain, particularly in understanding the complex interplay between cultural norms and sociodemographic factors. Addressing the public health implications of alcohol use necessitates sustained, high-quality research and the development of evidence-based strategies tailored to the unique cultural contexts of Spanish communities. Collaborative efforts with local stakeholders and community groups are essential to foster a culture of responsible alcohol consumption and mitigate associated risks. In this context, the present study aimed to examine alcohol consumption patterns within the adult population of Spain, identifying the sociodemographic variables, health habits, and lifestyle factors that influence such consumption.

## 2. Materials and Methods

### 2.1. Type of Study and Sampling

This research employed a cross-sectional study design with a quantitative approach. The sample included individuals over 18 years old residing in Spain. To avoid bias, individuals with chronic illnesses or temporary conditions potentially affecting their diet or those who could not voluntarily choose which foods and beverages to consume were excluded.

### 2.2. Ethical Requirements

The study adhered to the ethical guidelines of the Declaration of Helsinki [24] and received approval from the Research Ethics Committee of the Catholic University of Valencia (approval code UCV/2019–2020/152, 18 June 2020). Before questionnaire completion, participants were required to express their informed consent to participate in the study and to have the data collected used for the purpose of research.

### 2.3. Data Collection

The questionnaire was hosted on Google Forms and distributed through a non-probabilistic snowball sampling approach. This approach was chosen because it offers significant advantages, particularly in reaching large and diverse segments of the population in a short time and with limited resources. It is especially effective in collecting data on sensitive topics, such as alcohol consumption, where trust and social networks can facilitate participation. This method allowed us to obtain a wide sample of over 22,000 respondents, enhancing the overall richness and scope of the data. With Instagram serving as the primary platform to spread the survey, an Instagram account, @elretonutricional, was created specifically for this purpose, allowing various professionals, influencers, and supporters to assist in spreading the questionnaire. The researchers also used their networks on several social media such as LinkedIn, Twitter, WhatsApp, and Facebook.

The questionnaire was also distributed in physical format. Various shops and establishments across Spain—selected for the diversity of their clientele—were contacted and asked to display a poster in their premises. The poster provided information about the study and included a QR code linking directly to the online questionnaire, allowing customers to participate voluntarily. Additionally, an email was sent to a range of organizations operating throughout Spain (such as housewives’ associations, scout groups, and charitable entities), requesting their collaboration in sharing the survey with their members. Data collection took place from August 2020 to November 2021.

### 2.4. Measurements

The instrument used to gather detailed information on the nutritional, social, and lifestyle habits of the population, recording their choices or frequency of consumption, was the Nutritional and Social Healthy Habits scale (NutSo-HH) [25]. The instrument was previously developed following rigorous methodological approaches and psychometrically tested to ensure reliability and validity.

#### 2.4.1. Sociodemographic Variables

Sociodemographic variables include the participants’ sex (which was analyzed in binary form, distinguishing between male and female), age (which was categorized between young people who were 18–30 years old, and adults who were 31 years old or over), educational level (which was categorized into Basic education [no studies, only primary or secondary studies, vocational training or baccalaureate] and Higher education [degree, Master’s, and PhD]), and income level (categorized into three groups: low income (≤2200 EUR/month), medium–high income (>2200 EUR/month), and not specified). This threshold was defined based on socioeconomic standards relevant to Spain. According to Royal Decree 231/2020 of 4 February, the Minimum Individual Wage (Salario Mínimo Interprofesional, SMI) for 2020 was set at 950 EUR per month in 14 payments, equivalent to approximately 1108 EUR/month when divided into 12 payments. Considering Spain’s economic structure and the assumption that a household requires at least double the individual SMI to maintain an adequate living standard, we established 2200 EUR/month as the reference point for distinguishing low versus medium–high household income levels in this study [26], municipality (differentiating between municipalities with <2000 inhabitants, between 2000 and 10,000 inhabitants, and more than 10,000 inhabitants), living arrangement (living alone or living with others), family living (living in the family home or living outside), and place of residence (different regions of Spain).

#### 2.4.2. IASE Healthy Eating Index for the Spanish Population

The results from the food frequency variables were used to calculate the Healthy Eating Index for the Spanish population (IASE). We used a shortened, validated version by Norte and Ortiz [27], which includes key food categories such as “fruit”, “vegetables”, “meat”, “dairy”, “cereals”, “pulses”, and “soft drinks”. The IASE assesses the frequency of consuming foods recommended for daily or weekly intake, as well as those advised only occasionally. It also considers dietary diversity, an essential factor for a balanced diet. A score of 10 is awarded for dietary practices that align with the recommendations of the Spanish Society of Community Nutrition (SENC) [28], with a maximum score of 73. Based on the IASE score achieved, nutritional habits are grouped into three categories: “Healthy” for scores between 58.4 and 73, “Needs changes” for scores between 36.5 and 58.4, and “Unhealthy” for scores below 36.5.

#### 2.4.3. Potential Eating Disorders Variables

Variables related to potential indicators of eating disorders included worry about gaining weight or feeling overweight, difficulty controlling food intake or experiencing shame after eating, and concerns about body image. These frequency-based variables were assessed using a Likert scale ranging from 1 to 6, where 6 indicated “always”, 5 “very frequently”, 4 “frequently”, 3 “occasionally”, 2 “rarely”, and 1 “never”. Additionally, the survey specifically asked participants if they had ever been diagnosed with an eating disorder.

#### 2.4.4. Nutritional, Beverage Consumption, Health and Lifestyle Variables

Following the same criteria used in previous articles [29,30], reporting the detailed variable categorization, the nutritional variables that were not addressed by the IASE were categorized on a 4-point Likert scale. The same approach was applied to the lifestyle variables, except for body mass index (BMI) and minutes of sport, which were used as a numerical. In this scale, a rating of 1 signifies no or low frequency, while a rating of 4 corresponds to maximum frequency.

For the frequency of consumption of the different beverages, the categorization used was the same as in previous articles [31], and it is shown in detail in Table 1.

#### 2.4.5. Type of Alcohol Consumed

To explore the type of alcohol consumed by the population, the specific question asked was as follows: ‘*What types of alcoholic beverages do you usually consume (more than 2 times a month)?*’ Respondents were offered the possibility to choose one or more of the following categories:

- Fermented beverages (Wine, Champagne, Cava, Beer, Cider, Vermouth, Sake, etc.);
- Distilled beverages (Whisky, Vodka, Tequila, Rum, Gin, Pisco, Orujo, Brandy, Cognac, etc.);
- Fortified beverages (Fortified wine, Port, Sherry, Madeira, Manzanilla, Palomino, etc.);
- Liqueurs and creams (Liqueurs, Pacharán, Amaretto, Cream of orujo, etc.).

### 2.5. Data Analysis

The data collected were entered into a Microsoft Excel database and reviewed to correct any errors and inconsistencies, with special attention to entry issues and outliers. Some variables were categorized or calculated based on others. To ensure data reliability, extreme BMI values (below 14 and above 40) were excluded.

Once the data were organized and defined, they were transferred to Jamovi (Version 2.3.28.0) [32] for further analysis. The sample’s normality was assessed using the Shapiro–Wilk test, which indicated that none of the variables met normality assumptions., as confirmed by Q-Q plots [33]. The Chi-Square Test was then applied for categorical variables, while the Mann–Whitney U-test was chosen for independent ordinal or numeric variables. The significance level was set at 0.05. Discrete variables are presented as absolute values and percentages, and continuous variables are reported as mean and standard deviation. Additionally, the Dwass–Steel–Critchlow–Fligner pairwise comparison test was used to further analyze differences found between multiple groups or pairs.

To explore the conditional relationships between various types of alcohol and the consumption of other beverages, a network analysis approach was applied. In this model, each node (depicted as a circle) represents a specific variable, while the edges (lines connecting the nodes) illustrate the strength and direction of the associations between them. Edges are both color- and thickness-coded to convey correlation characteristics: green lines represent positive associations, whereas red lines indicate negative ones. Thicker lines correspond to stronger associations, while lighter hues—regardless of color—suggest weaker correlations.

The edge weights were derived using Spearman correlation coefficients. To isolate direct associations and minimize the influence of indirect or spurious relationships, the network was estimated using a Gaussian graphical model (GGM), selected through the Extended Bayesian Information Criterion (EBIC). This method allows for the computation of partial correlations by controlling for the influence of other variables, thereby improving the accuracy of the network representation.

Moreover, the inclusion of EBIC serves as a regularization mechanism, encouraging a more parsimonious and stable network structure and reducing the likelihood of overfitting. This type of visualization offers a comprehensive perspective on the multidimensional interactions within the dataset, enabling the identification of behavioral patterns, clusters of related variables, and possible compensatory dynamics between positively and negatively correlated components.

## 3. Results

### 3.1. Sociodemographic Characteristics

The sample includes 80.8% women, with a mean age of 34.9 years and high education level, with 68.3% holding a bachelor’s degree or higher. The distribution by income level was fairly even; 43.9% of respondents had a low-income level versus 47.9% who had a medium–high income, while 8.3% preferred not to answer the question. Further details of sample sociodemographic characteristics are presented in Table 2.

### 3.2. Analysis of the Alcohol Consumption

Regarding alcohol consumption, 10,686 respondents (48.2%) stated that they never or very sporadically consumed alcohol, 7330 (33.0%) consumed alcohol rarely (between 2 and 4 times a month), 3057 people (13.8%) reported that they drank alcohol between 2 and 3 times a week, while 1108 (5.0%) consumed alcohol more than 4 times a week or daily. Table 3 presents alcohol consumption patterns across various sociodemographic groups, both in the overall sample and separately for men and women. Overall, men reported significantly higher alcohol consumption (M = 1.97, SD = 0.99) compared to women (M = 1.71, SD = 0.83; *p* < 0.001).

Age-related differences were also observed. In the overall sample, adults (>30 years) consumed alcohol more frequently than younger participants aged 18–30 (M = 1.80 vs. 1.69; *p* < 0.001). When stratified by gender, this difference was especially pronounced among men (M = 2.13 vs. 1.74; *p* < 0.001), whereas no significant difference was observed among women (M = 1.72 vs. 1.68; *p* = 0.807).

Regarding education level, individuals with Higher education consumed more alcohol (M = 1.80) than those with only Basic education (M = 1.66; *p* < 0.001). This pattern held for both men and women, with the gender-specific differences reaching statistical significance in each subgroup (*p* < 0.001).

Income level also showed a significant effect: participants with medium to high incomes reported higher consumption (M = 1.84) compared to those with lower incomes (M = 1.68; *p* < 0.001). Again, this trend was consistent across genders, with men showing the most marked difference (M = 2.11 vs. 1.80; *p* < 0.001).

Household composition influenced drinking behavior as well. Participants living with family consumed slightly more alcohol than those not living with family (M = 1.83 vs. 1.73; *p* < 0.001). Among women, this difference was significant (*p* < 0.001), while among men, it was not (*p* = 0.304). On the other hand, when comparing individuals living alone versus those not living alone, women living alone drank significantly more (M = 1.82 vs. 1.69; *p* < 0.001), while no significant difference was found among men (*p* = 0.655).

These findings highlight distinct patterns of alcohol consumption across demographic groups, with notable gender-specific variations.

**Figure 1:**
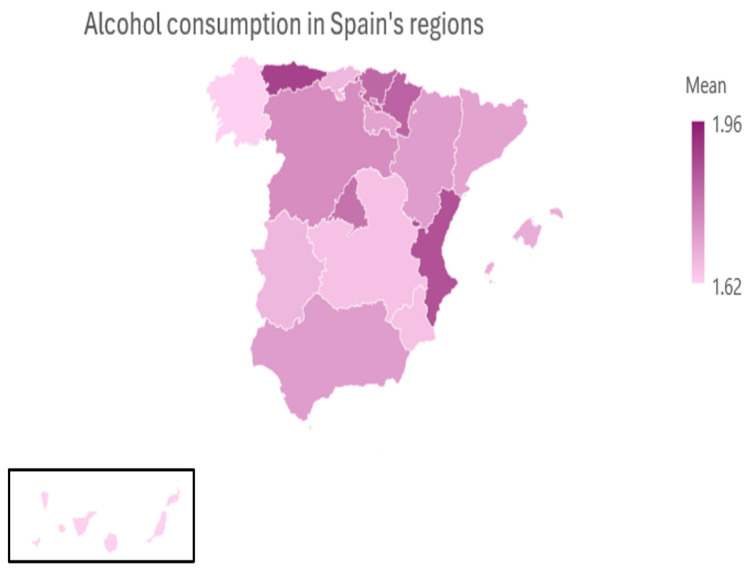
Comparison of alcohol consumption frequency differentiated by regions of Spain.

**Figure A1:**
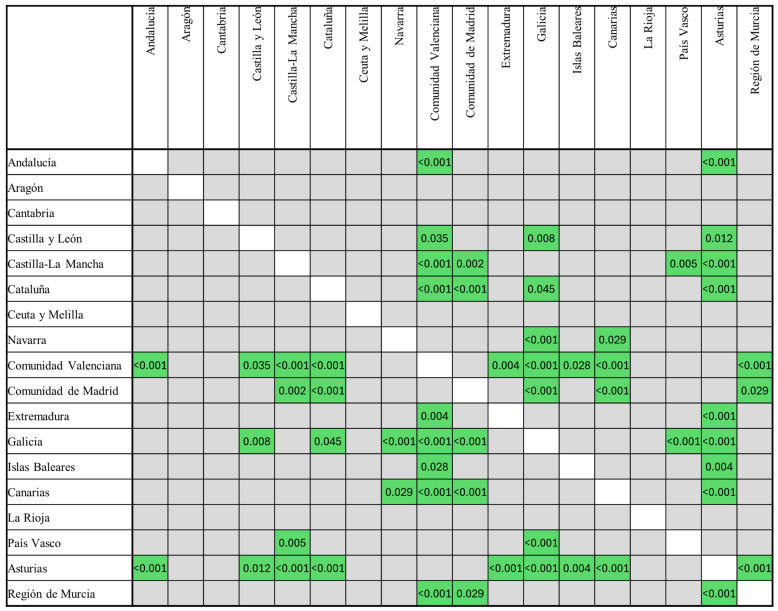
*p*-values of the frequency of alcohol consumption for regions of Spain compared using the Dwass–Steel–Critchlow–Fligner pairwise test. (NOTE: In the grey boxes, the *p*-value is not statistically significant >0.05, while the cells in green show statistically significant deviations).

Table 4 and Figure 1 present the average frequency of alcohol consumption across the 17 autonomous communities in Spain (Figure A1 in Appendix A shows in detail between which regions this difference is found). The data show modest but notable regional differences in reported alcohol use. The highest mean frequencies were observed in Ceuta and Melilla (M = 1.96, SD = 1.04), Asturias (M = 1.89, SD = 0.86), and the Valencian Community (M = 1.86, SD = 0.94), suggesting a relatively higher prevalence of regular alcohol consumption in these areas.

Other regions with above-average consumption included Navarra (M = 1.83), the Basque Country (M = 1.82), and the Community of Madrid (M = 1.80). In contrast, the lowest mean frequencies were reported in the Canary Islands (M = 1.62), Galicia (M = 1.62), Castilla-La Mancha (M = 1.65), and the Region of Murcia (M = 1.65), indicating more moderate or occasional drinking patterns.

Overall, while the variation in mean scores between regions is relatively small, the data reflect subtle geographical differences in alcohol consumption behavior across Spain. These findings may be influenced by cultural, social, and economic factors that warrant further investigation.

### 3.3. Analysis of the Type of Alcohol Consumed

**Figure 2:**
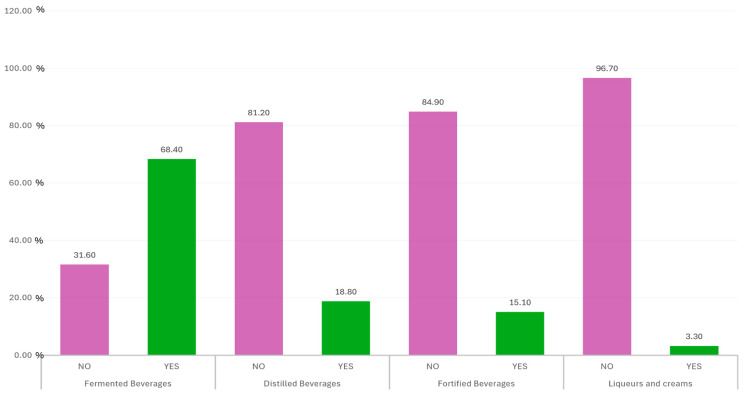
Analysis of type of alcohol consumed more than 2 times a month, with percentages of people drinking or not drinking a type of alcohol.

Looking in more detail at the type of alcohol consumed, the answers to the questions on the type of alcoholic beverage consumed are illustrated in Figure 2, where the numbers express the percentage of consumption or non-consumption of that type of alcoholic beverage.

Table 5 shows an analysis of the consumption of the different types of alcohol with respect to the sociodemographic variables of gender, age, level of education and income, size of municipality, living alone or accompanied, or living with the family or outside the family home. For fermented beverages, statistically significant differences are observed for all variables, while for the consumption of liqueurs and creams, there are no differences for any of them; finally, a mixed situation is observed for the consumption of distilled and fortified beverages.

Analyzing more specifically the sex variable, fermented beverages were the most commonly consumed, with 54.6% of women and 13.8% of men reporting consumption, although a significant portion of the sample (31.6%) reported no consumption at all, predominantly among women. Distilled beverages showed a lower overall consumption rate, with 81.2% of the sample indicating non-consumption—66.5% of them women and 14.7% men. Similarly, fortified beverages were consumed by only 15.2% of the sample, with 13.1% of women and 2.1% of men reporting intake. Liqueurs and creams had the lowest consumption rates across all categories, with 99.3% of the sample not consuming them; only 2.6% of women and 0.7% of men reported consumption. These findings suggest that while fermented beverages are relatively more common, especially among women, the consumption of other categories is limited across both sexes, with a slightly higher prevalence among women in most cases.

**Figure 3:**
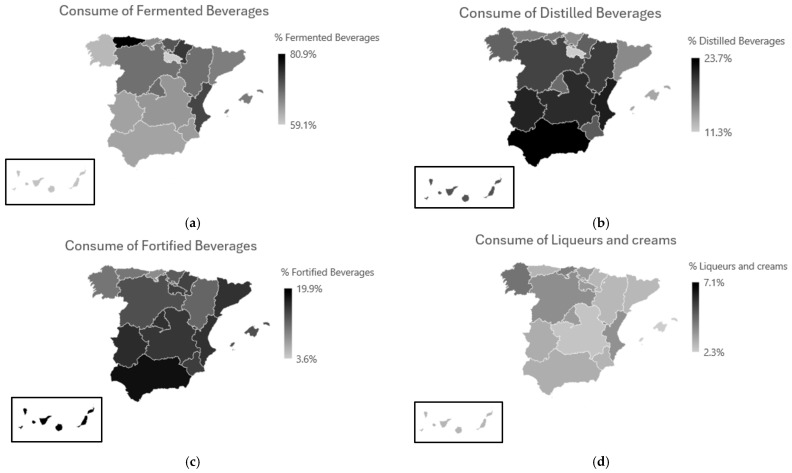
Analysis of the type of alcohol consumed by region in Spain. (**a**) Fermented beverages, (**b**) distilled beverages, (**c**) fortified beverages, and (**d**) liqueurs and creams.

Table 6 and Figure 3 provide a regional breakdown of alcohol consumption types across Spain, focusing on beverages consumed more than twice per month. Fermented beverages, including wine, beer, and cider, were the most commonly consumed type in all regions. Asturias led with the highest percentage of fermented beverage consumers (80.9%), followed by Navarra (74.7%), and the Valencian Community (73.3%). In contrast, the lowest prevalence was reported in La Rioja (59.1%) and the Canary Islands (60.3%).

Distilled beverage consumption, such as gin or whisky, showed more modest regional variation, with the highest consumption seen in the Valencian Community (21.6%) and Andalusia (23.7%), and the lowest in La Rioja (11.3%). Fortified wines, such as sherry and port, also exhibited regional disparities. Notably, the Canary Islands (19.9%) and Andalucía (18.2%) reported relatively higher consumption, aligning with traditional beverage preferences in those regions.

Liqueurs and creams were the least consumed across all regions, never exceeding 4.5% in any area. The highest prevalence was seen in Galicia (4.4%) and Cantabria (4.1%), while in several regions—such as the Balearic Islands and Castilla-La Mancha—consumption rates fell below 3%.

**Figure A2:**
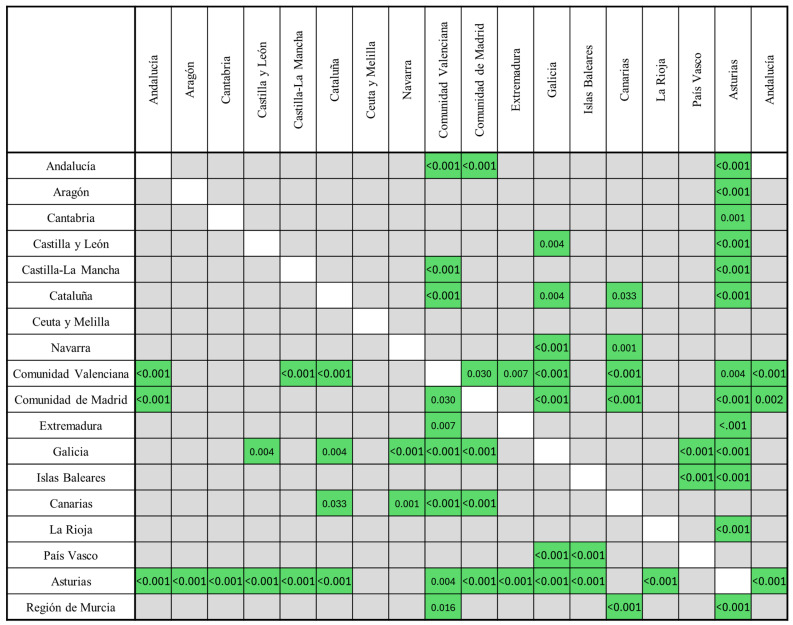
*p*-values of the frequency of fermented beverage consumption for regions of Spain compared using the Dwass–Steel–Critchlow–Fligner pairwise test. (NOTE: In the grey boxes, the *p*-value is not statistically significant >0.05, while the cells in green show statistically significant deviations).

**Figure A3:**
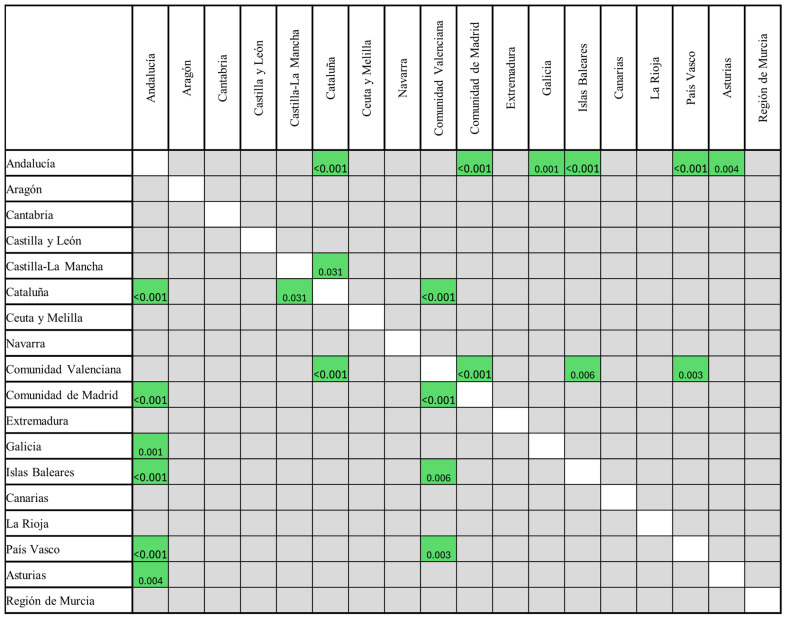
*p*-values of the frequency of distilled beverage consumption for regions of Spain compared using the Dwass–Steel–Critchlow–Fligner pairwise test. (NOTE: In the grey boxes, the *p*-value is not statistically significant >0.05, while the cells in green show statistically significant deviations).

**Figure A4:**
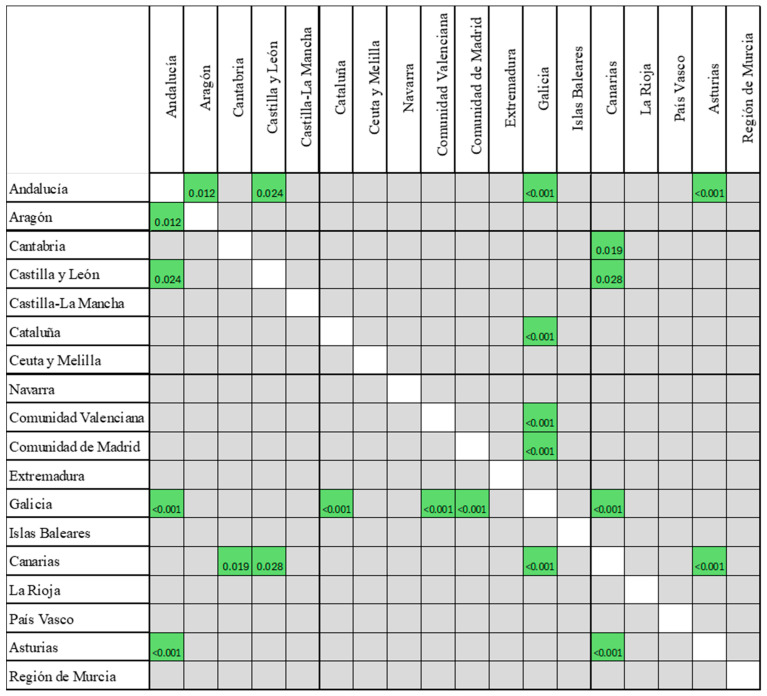
*p*-values of the frequency of fortified beverage consumption for regions of Spain compared using the Dwass–Steel–Critchlow–Fligner pairwise test. (NOTE: In the grey boxes, the *p*-value is not statistically significant >0.05, while the cells in green show statistically significant deviations).

Kruskal–Wallis tests confirmed statistically significant regional differences in the consumption of fermented (*p* < 0.001), distilled (*p* < 0.001), and fortified beverages (*p* < 0.001), while no significant differences emerged for liqueurs and creams. These results underscore the cultural and geographical influences on beverage preferences in Spain and highlight the need for tailored public health strategies. Figure A2, Figure A3 and Figure A4 in Appendix A show in detail between which regions this difference is found for the different types of alcoholic beverages.

### 3.4. Gaussian Graphical Model Between Different Types of Alcohol and Other Beverages

**Figure 4:**
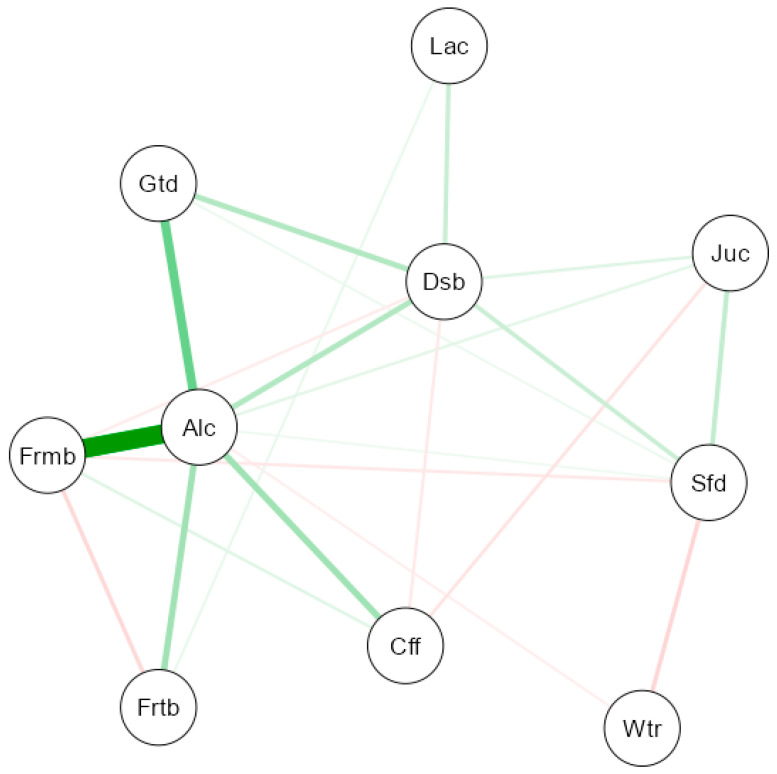
Gaussian graphical model between different types of alcohol and other beverages. (NOTE: The thickness of the lines reflects the magnitude of the relationship (partial correlation), while the colour indicates the direction: green for positive relationships and red for negative relationships).

**Figure A5:**
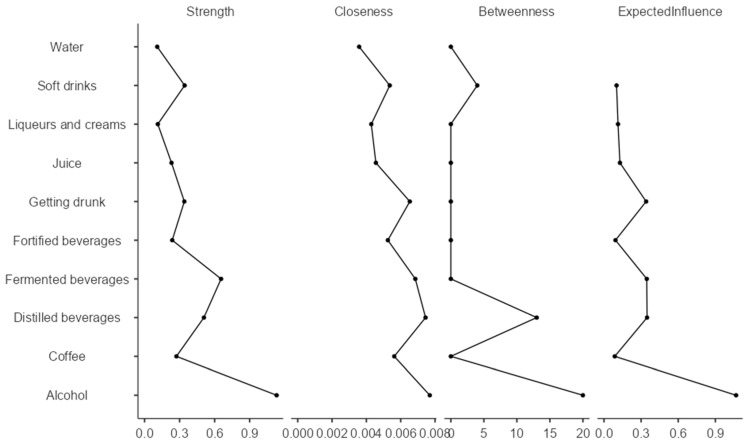
Centrality measures in network analysis. NOTE: Centrality metrics provide insight into the role and influence of each node within the network. Strength reflects the total magnitude of a node’s associations with all other nodes, regardless of direction; a higher strength value indicates a highly connected variable. Closeness assesses how easily a node can reach or be reached by others across the network; high closeness suggests that the variable can rapidly influence—or be influenced by—others through relatively short paths. Betweenness captures how often a node lies on the shortest path between two other nodes, identifying variables that act as key intermediaries or bridges within the network, even if they have few direct connections. Expected influence is similar to strength but accounts for the direction (positive or negative) of the relationships, offering a more nuanced view of each node’s overall impact on the network.

**Figure A6:**
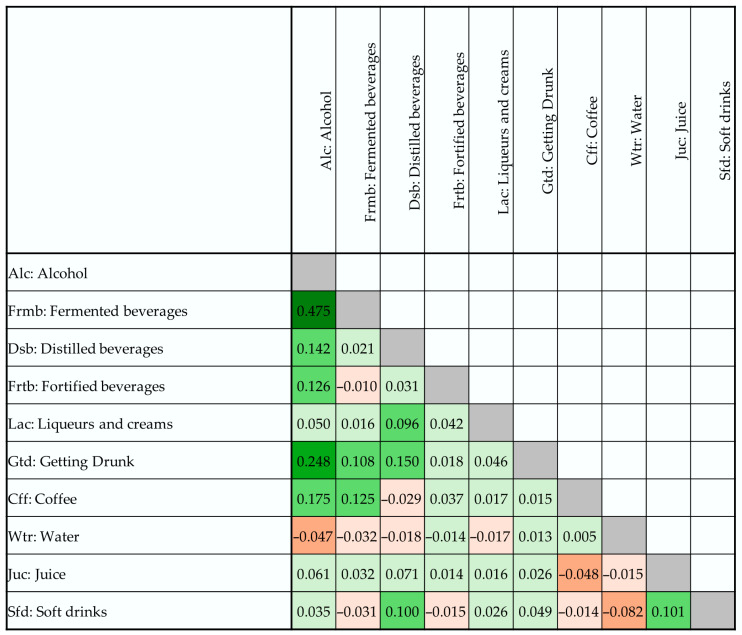
Values in network analysis. (NOTE: Green cells represent positive associations, whereas red cells indicate negative ones, while the colour intensity represents the degree of intensity of the relationship between the pair of variables considered in each cell).

Finally, a Gaussian graphical model was created (Figure 4) to identify the conditional relationships between different types of alcohol and the consumption of other beverages (water, coffee, sugary drinks, juice) in the Spanish population. Figure A5 in Appendix B shows the centrality measures of the network analysis for the different variables of the study, while Figure A6 shows the associations between the different variables.

In Figure 4, it can be observed that the node corresponding to alcohol (Alc) occupies a central position, standing out due to the number and thickness of its connections with other types of beverages, which highlights its key role in consumption patterns.

The most notable connection is observed between alcohol (Alc) and fermented beverages (Frmb), with a thick and intense green edge that suggests a strong positive conditional correlation. This reflects a direct relationship between the consumption of alcoholic and fermented drinks, which is expected since many fermented beverages (such as beer or wine) are considered part of the alcoholic group, and their joint or alternating consumption is common in Spain. A relevant association is also seen between alcohol (Alc) and distilled beverages (Dsb), indicating that those who generally consume alcoholic drinks also tend to consume distilled ones, such as whiskey, rum, or vodka. This relationship confirms the coexistence of different types of alcoholic beverages within usual consumption patterns.

The link with getting drunk (Gtd) is equally important. The positive connection suggests that overall alcohol consumption is strongly related to the habit of getting drunk, which raises a public health concern.

Other positive but more moderate connections appear between alcohol and drinks such as fortified beverages (Frtb), coffee (Cff), and liqueurs and creams (Lac). The association with fortified beverages may indicate more traditional or social consumption patterns. In the case of coffee and liqueurs/creams, this may reflect situations of joint consumption in social settings or after meals.

On the other hand, the model shows negative connections (red edges) between alcohol and some beverages considered healthier or more common in daily consumption, such as water (Wtr) and soft drinks (Sfd). Although these relationships are weaker, they could suggest that in certain consumer profiles, higher alcohol intake is associated with lower intake of these beverages. In particular, the negative relationship with water may be relevant in terms of hydration and overall health. Lastly, the connection with juice (Juc) is weakly positive, which might reflect a certain coexistence of alcohol with beverages like juices, possibly in cocktails or social events.

## 4. Discussion

Alcohol consumption in Spain is a complex phenomenon influenced by a variety of factors, including personal preferences, social dynamics, and cultural traditions. The analysis of sociodemographic, behavioral, and cultural variables provides a rich and multifaceted overview, highlighting the diverse nature of alcohol-related experiences in the country.

The study, conducted on a large sample of 22,181 individuals, revealed how gender, age, level of education, income, and housing context, as well as specific regional differences, are important determinants for understanding consumption behavior.

Men consume more alcohol than women on average, in line with findings by Kerr-Corrêa et al. [34], who attribute this difference to biological, social, and cultural factors. Biologically, men and women metabolize alcohol differently due to variations in body composition and enzymatic activity. Women generally have lower total body water content and reduced activity of alcohol dehydrogenase, leading to higher blood alcohol concentrations for equivalent alcohol intake [35]. These physiological differences may contribute to more cautious consumption behaviors among women. From a sociocultural perspective, traditional gender norms and social expectations often frame alcohol use as more acceptable for men, while women may experience greater stigma for similar behaviors. However, changing gender roles and increased targeted marketing toward women have led to narrowing gender gaps in some regions [36,37]. The female predominance in the sample (80.8%) probably reflects a greater propensity of women to participate in public health studies, a phenomenon already observed in previous research. Ruiz et al. [38] highlighted that usually women tend to respond more frequently to surveys and questionnaires, especially on health-related topics.

The mean sample age of 34.9 years, with slightly older men (36.5) compared to women (34.5), reflects a demographic where alcohol consumption often peaks. This trend aligns with patterns observed in Spain, where alcohol is culturally embedded in social and culinary practices, promoting regular but moderate intake among adults [39]. Younger populations, in contrast, tend to favor more episodic and heavy drinking, often in social or festive contexts [40]. Conversely, studies from North America have found a decline in alcohol consumption with increasing age, often attributed to shifting life responsibilities and heightened health awareness [37]. These contrasting patterns underscore the role of sociocultural context in shaping age-related drinking behaviors and the importance of localized public health strategies.

An interesting aspect concerns the level of education, which was above average, with 68.3% of the sample having completed advanced studies. Although the literature often links higher education to greater awareness of alcohol-related risks and, sometimes, to more moderate consumption [38], in this case, a different relationship was observed. A higher level of education was associated with more frequent consumption, in line with findings by Kerr-Corrêa et al. [34], who highlighted how education can be linked to greater socialization in contexts involving alcohol consumption.

Income also played a significant role. Respondents with medium–high income (47.9%) showed a higher propensity to consume alcohol regularly than those with low income (43.9%). Anderson et al. [41] attributed this phenomenon to greater accessibility to alcohol and the frequency of social events among higher-income groups. However, as noted by Garcia and Ramos [42], lower-income groups can exhibit significant consumption, often linked to cheaper forms of alcohol, with potential negative consequences for health.

Family and social dynamics emerge as another key factor. Those living with family or in cohabitation have a higher average consumption (1.83 and 1.85) than those living alone (1.75). Ruiz et al. [38] attribute this to the social and convivial nature of alcohol consumption in Mediterranean cultures, where drinking is often part of family occasions. In Mediterranean cultures, alcohol consumption is often integrated into daily life, particularly within family and cohabiting households. This integration is characterized by moderate wine consumption during meals and social gatherings, contributing to a lifestyle associated with longevity and reduced stress. The social aspect of drinking in these settings fosters community bonds and may lead to more mindful consumption patterns [43]. However, Villar et al. [44] warn that those who live alone, despite consuming less alcohol, may be more vulnerable to episodes of abuse related to loneliness. Individuals living alone may experience increased psychological distress, such as loneliness and reduced self-esteem, which can influence alcohol consumption patterns. Studies have shown that loneliness is associated with problematic alcohol use, and self-esteem mediates this relationship. This suggests that interventions aimed at improving self-esteem and reducing loneliness could be effective in addressing alcohol misuse among those living alone [45].

Geographically, the distribution of the sample shows a prevalence of residents in urban areas (79.3%), with a greater concentration in the south of Spain. Anderson et al. [41] highlighted that urban areas tend to favor higher consumption due to the greater availability of alcohol and an intense social life. Regional differences, with average consumption ranging between 1.62 and 1.96, reflect local traditions and specific health policies. Regions such as Ceuta, Melilla, and Asturias stand out for higher average consumption, attributable to traditions such as the widespread consumption of cider in Asturias (80.94%), while regions such as Galicia and the Canary Islands show lower consumption, probably linked to a lesser cultural rootedness of alcohol or a greater sensitivity to its risks [41].

Finally, preference for fermented beverages (68.4%) over spirits (18.8%) or liqueurs (3.3%) confirms the predominance of wine and beer, typical of southern Europe. Pérez and Torres [46] highlighted how these beverages are more integrated into food traditions than spirits. These differences also reflect the varying degrees of social acceptance and perception of risk associated with the various categories of alcohol, with beer and wine perceived as “safer” than spirits, as observed by González and Ortega [47]. The perceived risk associated with different types of alcoholic beverages varies. Wine is often perceived as a safer option due to its association with meals and its lower alcohol content. In contrast, spirits are viewed as riskier, partly due to their higher alcohol content and the drinking behaviors associated with their consumption. This perception influences consumption choices and may contribute to the preference for wine and beer over spirits [48]. Cultural norms also influence alcohol consumption preferences. In Mediterranean countries, wine and beer are deeply embedded in social and culinary traditions, leading to their higher consumption compared to spirits. These cultural practices promote regular but moderate consumption patterns, contrasting with the binge-drinking behaviors more commonly associated with spirits in other regions [49,50].

The results of the study suggest some global trends, including the association between higher income and greater alcohol consumption. As Collins [51] highlight, this dynamic is particularly pronounced in urban contexts, where social status significantly influences consumption habits. However, unique characteristics emerge that distinguish Spain. While in other European countries, alcohol consumption is more widespread among lower-income groups, in Spain, consumption appears to be closely linked to higher social status. This phenomenon translates into a tendency toward regular but moderate consumption among adults, in stark contrast to the binge-drinking patterns frequently observed in other European regions. Rodriguez-Sanchez et al. [52] explored these polarizations in drinking behaviors, highlighting how binge-drinking is more common elsewhere, while in Spain, alcohol consumption is more harmoniously integrated into social routines.

Although the study data confirm many of the trends already noted in the literature, they offer insights into dynamics that are more specific to the Spanish context. For example, global studies such as those by Anderson, Baumberg, and Rossow [41] have documented a decline in alcohol consumption among young adults in some areas of the world. This trend is reflected in Spain, where a significant presence of abstinent or occasional consumers is observed. What makes the Spanish case unique, however, is the association between middle–high income and regular alcohol consumption, a relationship that, according to Rodríguez and Sánchez [52], differs from trends observed in other European countries, where lower-income groups often show higher consumption. This suggests a particular cultural relationship with alcohol consumption, deeply rooted in social status.

Furthermore, the role of the family and social context emerges as a central element. As Delgado and Álvarez [53] point out, alcohol consumption in Spain, and more generally in Mediterranean countries, is strongly linked to convivial moments and cultural traditions. This peculiarity differentiates Spain from Nordic or Anglo-Saxon contexts, where alcohol consumption tends to be less associated with social occasions and more frequently characterized by episodes of heavy consumption [54]. In Spain, however, moderate and regular consumption, often linked to meals or family events, represents an integral part of the local culture, offering a starting point to better understand the differences in alcohol patterns between regions and countries.

### 4.1. Network Analysis Results

The network analysis results provide a clear overview of alcohol consumption patterns in relation to other beverages and behaviors within the Spanish population. The strong associations with fermented and distilled beverages, as well as with episodes of intoxication, highlight an urgent need for public policies aimed at reducing excessive alcohol intake and encouraging healthier habits. Simultaneously, the negative associations with water and soft drinks may indicate valuable opportunities to promote these alternatives as substitutes for alcohol in social settings. These findings can also inform the design of targeted educational campaigns, particularly for youth and high-risk groups, promoting moderation in alcohol consumption and raising awareness of its potential health consequences.

The strong connection between alcohol and the variable “getting drunk” (Gtd) underscores a concerning pattern of use that may go beyond occasional or moderate intake, especially among certain subgroups of the population. Additionally, the co-occurrence of alcohol with beverages like coffee, liqueurs/creams, and fortified drinks may reflect culturally embedded consumption routines—such as post-meal drinking or festive social practices—that should be considered when designing culturally sensitive interventions. These associations also suggest that alcohol is often consumed alongside other stimulant or sweetened beverages, possibly reinforcing unhealthy routines.

Of particular interest are the negative links between alcohol and both water and soft drinks, which may indicate mutually exclusive consumption preferences. These patterns could guide future health promotion strategies, encouraging increased water consumption as a replacement for alcoholic drinks, particularly in leisure contexts. Moreover, the central position of alcohol in the network suggests it acts as a behavioral hub within the broader lifestyle habits of the Spanish population, reinforcing its relevance as a focal point for public health initiatives.

In summary, network analysis not only confirms expected associations but also reveals less intuitive relationships that can serve as the basis for more nuanced, multifaceted public health approaches. It highlights the need to address alcohol consumption not in isolation, but as part of a complex system of inter-related dietary and social behaviors.

### 4.2. Strengths and Limitations

Among the most relevant aspects of this study, the multidimensional approach stands out, exploring a wide range of sociodemographic variables, such as age, gender, level of education, and economic status. This method allows researchers to outline a complex and articulated picture of consumption habits, providing a deeper and more detailed understanding. Furthermore, the research stands out for its cultural relevance: it does not limit itself to quantitatively describing alcohol consumption but also delves into the symbolic and cultural meanings associated with different beverages. This perspective enriches the analysis, allowing for the investigation of social and identity dynamics that influence consumer behavior. The breadth of the sample with 22,181 Spanish adults is undoubtedly an important strength of the study.

Despite these strengths, the study has some critical issues. A limitation of this study pertains to the sampling approach, which primarily employed snowball sampling and relied extensively on social media for distribution. Although snowball sampling enables the collection of diverse responses, it is also susceptible to self-selection bias since the recruitment of participants depends on initial respondents recommending others. On social media, influencers play a crucial role in disseminating content on specific topics, often attracting followers with shared interests and behaviors. Consequently, it is plausible that the survey reached certain communities influenced by these dynamics, potentially shaping participants’ behaviors and preferences. To mitigate these biases, significant efforts were made to expand the distribution of the questionnaire beyond social networks. In particular, the survey was also shared in physical form to reach a broader audience.

One of the most prominent biases in the sample was gender imbalance, with approximately 80% of respondents being female. Despite concerted efforts by the authors to achieve a more balanced sample, this bias persisted and may be inherent to research of this nature. Similar trends have been observed in other studies on related topics, where women demonstrate a greater willingness to participate in research on nutrition and health [55,56,57].

Another bias consists of the variables used; despite including different sociodemographic groups, the analysis tends to neglect important variables, such as ethnicity, religious orientation, or other cultural factors, which could offer an additional key to understanding consumption habits. Another limitation is the lack of a longitudinal approach: the data come from cross-sectional analyses, which do not allow for the observation of behavioral changes over time or the capture of evolutions in consumption patterns. Finally, there is a risk of interpreting some consumption patterns in an overly rigid way, with the danger of stereotyping behaviors and neglecting individual exceptions or emerging dynamics that could further enrich the analysis.

In addition, the sample’s representativeness must be interpreted with caution. Although the total number of respondents is high, the demographic profile—mainly women with a relatively high educational level—does not fully reflect the broader Spanish population. This imbalance may limit the generalizability of the findings. However, the large sample size provides a degree of robustness and allows for the exploration of significant trends within this specific population group. To address this limitation, future studies should aim to implement stratified or probabilistic sampling strategies that ensure a more balanced representation of gender, education level, and other relevant sociodemographic variables. In fact, a new data collection phase has already been initiated with the goal of reaching a more demographically diverse sample, which will allow for further validation and comparison of the present findings.

### 4.3. Areas for Further Research

Future developments in alcohol consumption research could focus on several promising directions to deepen and expand current knowledge. A longitudinal trend analysis would be a crucial step to monitor the evolution of consumption patterns over time, linking cultural, economic, and regulatory changes with population drinking habits. This approach would allow researchers to understand how social changes influence consumer behavior, providing a basis for more targeted and timely interventions.

The exploration of additional variables could enrich the interpretative framework. Factors such as ethnicity, religious affiliation, or food preferences, currently little explored, could prove crucial in understanding the dynamics of alcohol consumption. In parallel, a study on the impact of public policies would be of great value to evaluate the effectiveness of alcohol regulations, awareness campaigns, and fiscal measures in changing consumer behavior and promoting more informed choices.

Qualitative approaches, such as interviews and focus groups, could complement quantitative analyses by offering a richer perspective on the cultural and symbolic meanings attributed to alcohol consumption. Such methods would allow for a deeper investigation of the personal and collective motivations that influence consumption choices, going beyond numerical data. In parallel, analyzing the effectiveness of interventions and educational programs aimed at specific sociodemographic groups could provide valuable insights on how to prevent and reduce the risks associated with problematic consumption.

Another relevant research direction could involve comparisons with other European countries to identify similarities and differences in consumption patterns. This international perspective would offer a broader view of alcohol dynamics, facilitating the development of shared strategies that can be adapted to different cultural contexts. Finally, the impact of technologies and new consumption models deserves particular attention. With the advent of socialization apps and digital platforms, which increasingly promote the consumption of alcoholic beverages, it would be interesting to analyze how these influence consumption practices and redefine their contexts and methods. These areas of research represent significant opportunities to broaden our understanding of the phenomenon and to develop more effective prevention and intervention strategies.

Finally, future research could address additional issues such as the cost of alcohol and outlet density by adopting a longitudinal approach to analyze transformations over time. A better balance between detailed analysis and an integrative perspective would allow for more comprehensive and representative results.

## 5. Conclusions

The study of alcohol consumption in Spain highlights a complex phenomenon influenced by cultural traditions, sociodemographic factors, and regional differences. A key finding is the moderate and regular consumption of alcohol deeply embedded in Spain’s social and culinary practices, contrasting with the binge-drinking patterns seen in northern countries. This consumption is often integrated into social and familial settings, reflecting the Mediterranean cultural heritage of moderation and conviviality.

The research underscores the importance of cultural influences on drinking behaviors, emphasizing Spain’s distinct patterns, such as the close relationship between alcohol consumption and social status. While confirming global trends, the study reveals unique dynamics in Spain’s alcohol consumption practices.

Future research should address the limitations of this study, including the need for longitudinal data and the exploration of additional variables such as ethnicity and public policy. This will help develop more targeted strategies for promoting responsible alcohol consumption in Spain and similar contexts.

## Figures and Tables

**Table 1:** Categorization used for the frequency of consumption of different beverages.

Variable	Category	Score
Water	Never and very rarely (2 max. per month) and 1 glass/cup/week and ≥2 glasses/cups/week	1
	2 glasses/cups or less every day	2
	3–5 glasses every day	3
	More than 5 glasses every day	4
Sugary soft drinks, coffee, and energy drinks	Never and very rarely (2 glasses max. per month)	4
	One glass per week and 2 or more glasses per week	3
	2 glasses or less every day	2
	3–5 glasses and ≥5 glasses every day	1
Juice	Never and very rarely (2 glasses max. per month)	1
	One glass per week and 2 or more glasses per week	2
	2 glasses or less every day	3
	3–5 glasses and ≥5 glasses every day	4
Alcohol consumption	Never or once a month	1
	2–4 times a month	2
	2–3 times a week	3
	4–5 times a week or every day	4

**Table 2:** Sample sociodemographic characteristics (N = 22,181).

		Mean (SD) or N (%)
Sex	Male	4251 (19.2%)
	Female	17,930 (80.8%)
Age in years	Age total (years)	34.9 (11.7)
	Age male (years)	36.5 (13.4)
	Age female (years)	34.5 (11.2)
Education level	Basic education	7027 (31.7%)
	Higher education	15,154 (68.3%)
Income level	Low	9727 (43.9%)
	Medium–high	10,616 (47.9%)
	No answer	1839 (8.3%)
Municipality	<2000	1014 (4.6%)
	2000–10,000	3587 (16.2%)
	>10,000	17,580 (79.3%)
Living arrangement	Living alone	2202 (9.9%)
	Not living alone	19,979 (90.1%)
Family life	Living with family	16,732 (75.4%)
	Living without family	5449 (24.6%)
Living area	North of Spain	9602 (43.3%)
	South of Spain	12,579 (56.7%)
Spanish communities	Andalusia	2617 (11.8%)
	Aragon	652 (2.9%)
	Asturias	661 (3.0%)
	Balearic Islands	488 (2.2%)
	Basque Country	925 (4.2%)
	Canary Islands	637 (2.9%)
	Cantabria	217 (1.0%)
	Castilla-La Mancha	729 (3.3%)
	Castile and León	1276 (5.8%)
	Cataluña	3540 (16.0%)
	Ceuta and Melilla	28 (0.1%)
	Community of Madrid	3882 (17.5%)
	Extremadura	395 (1.8%)
	Galicia	1404 (6.3%)
	La Rioja	115 (0.5%)
	Navarre	324 (1.5%)
	Region of Murcia	417(1.9%)
	Valencian Community	3874 (17.5%)

**Table 3:** Comparison of alcohol consumption differentiated by sociodemographic variables.

All Sample		*p*-Value ^$^	Only Men (N = 4251)		*p*-Value ^$^	Only Women		*p*-Value ^$^
**Male**	**Female**							
1.97 (0.99)	1.71 (0.83)	<0.001						
**Young** **(18–30 years)**	**Adults** **(>30 years)**		**Young** **(N = 1835)**	**Adults (N = 2416)**		**Young** **(N = 7857)**	**Adults** **(N = 10,073)**	
1.69 (0.78)	1.80 (0.94)	<0.001	1.74 (0.81)	2.13 (1.07)	<0.001	1.68 (0.77)	1.72 (0.88)	0.807
**Basic education**	**Higher education**		**Basic education (N = 1539)**	**Higher education** **(N = 2712)**		**Basic education** **(N = 5488)**	**Higher education** **(N = 12,442)**	
1.66 (0.85)	1.80 (0.88)	<0.001	1.83 (0.96)	2.05 (0.99)	<0.001	1.61 (0.81)	1.75 (0.84)	<0.001
**Low incomes**	**Medium–high incomes**		**Low incomes** **(N = 1646)**	**Medium–high incomes** **(N = 2305)**		**Low incomes** **(N = 8081)**	**Medium–high incomes** **(N = 8311)**	
1.68 (0.84)	1.84 (0.91)	<0.001	1.80 (0.93)	2.11 (1.02)	<0.001	1.65 (0.81)	1.77 (0.86)	<0.001
**Living with family**	**Living without family**		**Living with family** **(N = 3230)**	**Living without family (N = 1021)**		**Living with family** **(N = 13,502)**	**Living without family** **(N = 4428)**	
1.83 (0.86)	1.73 (0.87)	<0.001	1.98 (1.01)	1.92 (0.91)	0.304	1.67 (0.83)	1.81 (0.85)	<0.001
**Living alone**	**Not living alone**		**Living alone** **(N = 459)**	**Not living alone (N = 3792)**		**Living alone** **(N = 1743)**	**Not living alone** **(N = 16,187)**	
1.75 (0.87)	1.85 (0.90)	<0.001	1.94 (0.97)	1.97 (0.99)	0.655	1.82 (0.87)	1.69 (0.83)	<0.001

^$^ Mann–Whitney U-test.

**Table 4:** Comparison of alcohol consumption frequency differentiated by regions of Spain.

Spanish Regions	Mean (SD)
Andalucía	1.72 (0.86)
Aragón	1.72 (0.87)
Cantabria	1.67 (0.80)
Castilla y León	1.75 (0.86)
Castilla-La Mancha	1.65 (0.79)
Cataluña	1.71 (0.86)
Ceuta y Melilla	1.96 (1.04)
Navarra	1.83 (0.86)
Comunidad Valenciana	1.86 (0.94)
Comunidad de Madrid	1.8 (0.88)
Extremadura	1.67 (0.86)
Galicia	1.62 (0.83)
Islas Baleares	1.69 (0.85)
Canarias	1.62 (0.74)
La Rioja	1.71 (0.93)
País Vasco	1.82 (0.87)
Asturias	1.89 (0.86)
Región de Murcia	1.65 (0.83)

**Table 5:** Analysis of the consumption of the different types of alcohol with respect to the sociodemographic variables.

		**Fermented Beverages N (%)**		***p*-Value ^$^**	**Distilled Beverages N (%)**		***p*-Value ^$^**
		**NO**	**YES**		**NO**	**YES**	
Sex	Male	1186 (27.9%)	3065 (72.1%)	<0.001	3262 (76.7%)	989 (23.3%)	<0.001
	Female	5826 (32.5%)	12,104 (67.5%)		14,758 (82.3%)	3172 (17.7%)	
Age	Young (18–30 years)	3254 (14.7%)	6438 (29.0%)	<0.001	7323 (33.0%)	2369 (10.7%)	<0.001
	Adults (>30 years)	3758 (16.9%)	8731 (39.4%)		10,697 (48.2%)	1792 (8.1%)	
Education	Basic education	2599 (37.0%)	4428 (63.0%)	<0.001	5481 (78.0%)	1546 (22.0%)	<0.001
	Higher education	4413 (29.1%)	10,741 (70.9%)		12,539 (82.7%)	2615 (17.3%)	
Incomes	Low	3337 (16.4%)	6390 (31.4%)	<0.001	7948 (39.1%)	1466 (7.2%)	0.868
	Medium–high	3064 (15.1%)	7552 (37.1%)		8684 (42.7%)	1932 (9.5%)	
Municipality	<2000	1304 (5.9%)	2283 (10.3%)	<0.001	2903 (13.1%)	684 (3.1%)	0.647
	2000–10,000	378 (1.7%)	636 (2.9%)		815 (16.8%)	199 (0.9%)	
	>10,000	5330 (24.0%)	12,250 (55.2%)		14,302 (64.5%)	3278 (14.8%)	
Living arrangement	Living alone	6423 (29.0%)	13,556 (61.1%)	<0.001	16,232 (73.2%)	3747 (16.9%)	0.958
	Not living alone	589 (2.7%)	1613 (7.3%)		1788 (8.1%)	414 (1.9%)	
Family life	Living with family	1535 (6.9%)	3914 (17.6%)	<0.001	4262 (19.2%)	1187 (5.4%)	<0.001
	Living without family	5477 (24.7%)	11,255 (50.7%)		13,758 (62.0%)	2974 (13.4%)	
		**Fortified Beverages N (%)**		***p*-Value ^$^**	**Liqueurs and Creams N (%)**		***p*-Value ^$^**
		**NO**	**YES**		**NO**	**YES**	
Sex	Male	3789 (89.1%)	462 (10.9%)	<0.001	4101 (96.5%)	150 (3.5%)	0.316
	Female	15,032 (83.8%)	2898 (16.2%)		17,352 (96.8%)	578 (3.2%)	
Age	Young (18–30 years)	8352 (37.7%)	1340 (6.0%)	<0.001	9362 (42.2%)	330 (1.5%)	0.366
	Adults (>30 years)	10,469 (47.2%)	2020 (9.1%)		12,091 (54.5%)	398 (1.8%)	
Education	Basic education	6006 (85.5%)	1021 (14.5%)	0.08	6756 (96.1%)	271 (3.9%)	0.001
	Higher education	12,815 (84.6%)	2339 (15.4%)		14,697 (97.0%)	457 (3.0%)	
Incomes	Low	8261 (40.6%)	1466 (7.2%)	0.601	9385 (46.1%)	342 (1.7%)	0.053
	Medium–high	8988 (44.2%)	1628 (8.0%)		10,294 (50.6%)	322 (1.6%)	
Municipality	<2000	3037 (13.7%)	550 (2.5%)	0.179	3458 (15.6%)	129 (0.6%)	0.378
	2000–10,000	881 (4.0%)	133 (0.6%)		977 (4.4%)	37 (0.2%)	
	>10,000	14,903 (67.2%)	2677 (12.1%)		17,018 (76.7%)	562 (2.5%)	
Living arrangement	Living alone	16,999 (76.6%)	2980 (13.4%)	0.004	19,310 (87.1%)	669 (3.0%)	0.094
	Not living alone	1822 (8.2%)	380 (1.7%)		2143 (9.7%)	59 (0.3%)	
Family life	Living with family	4575 (20.6%)	874 (3.9%)	0.035	5259 (23.7%)	190 (0.9%)	0.329
	Living without family	14,246 (64.2%)	2486 (11.2%)		16,194 (73.0%)	538 (2.4%)	

^$^ Mann–Whitney U-test.

**Table 6:** Analysis of the type of alcohol consumed by region in Spain.

Regions	Fermented Beverage		Distilled Beverage		Fortified Beverage		Liqueurs and Creams	
	N	%	N	%	N	%	N	%
Andalucía	1666	63.66%	621	23.73%	476	18.19%	79	3.02%
Aragón	451	69.17%	130	19.94%	77	11.81%	18	2.76%
Cantabria	144	66.36%	36	16.59%	19	8.76%	9	4.15%
Castilla y León	881	69.04%	249	19.51%	172	13.48%	48	3.76%
Castilla-La Mancha	474	65.02%	152	20.85%	112	15.36%	18	2.47%
Cataluña	2394	67.63%	545	15.40%	557	15.73%	98	2.77%
Ceuta y Melilla	20	71.43%	4	14.29%	1	3.57%	2	7.14%
Navarra	242	74.69%	58	17.90%	47	14.51%	9	2.78%
Comunidad Valenciana	2840	73.31%	838	21.63%	612	15.80%	143	3.69%
Comunidad de Madrid	2701	69.58%	671	17.28%	615	15.84%	134	3.45%
Extremadura	252	63.80%	84	21.27%	64	16.20%	12	3.04%
Galicia	862	61.40%	249	17.74%	149	10.61%	62	4.42%
Islas Baleares	332	68.03%	67	13.73%	66	13.52%	11	2.25%
Canarias	384	60.28%	119	18.68%	127	19.94%	18	2.83%
La Rioja	68	59.13%	13	11.30%	17	14.78%	4	3.48%
País Vasco	664	71.78%	142	15.35%	117	12.65%	32	3.46%
Asturias	535	80.94%	107	16.19%	70	10.59%	19	2.87%
Región de Murcia	269	64.51%	76	18.23%	62	14.87%	12	2.88%

## Data Availability

The data presented in this study are available on request from the corresponding author due to privacy and ethical restrictions.

## Abbreviations

Alc	Alcohol
Frmb	Fermented beverages
Dsb	Distilled beverages
Frtb	Fortified beverages
Lac	Liqueurs and creams
Gtd	Getting drunk
Cff	Coffee
Wtr	Water
Juc	Juice
Sfd	Soft drinks
